# The appendicular morphology of *Sinoburius lunaris* and the evolution of the artiopodan clade Xandarellida (Euarthropoda, early Cambrian) from South China

**DOI:** 10.1186/s12862-019-1491-3

**Published:** 2019-08-06

**Authors:** Xiaohan Chen, Javier Ortega-Hernández, Joanna M. Wolfe, Dayou Zhai, Xianguang Hou, Ailin Chen, Huijuan Mai, Yu Liu

**Affiliations:** 1 Yunnan Key Laboratory for Palaeobiology, Yunnan University, North Cuihu Road 2, Kunming, 650091 China; 2 MEC International Joint Laboratory for Palaeobiology and Palaeoenvironment, Yunnan University, Kunming, 650091 China; 3 Museum of Comparative Zoology and Department of Organismic and Evolutionary Biology, Harvard University, 26 Oxford Street, Cambridge, MA 02138 USA; 4 Research Center of Paleobiology, Yuxi Normal University, Yuxi, 653100 Yunnan China

**Keywords:** Euarthropoda, Konservat-Lagerstätte, Exceptional preservation, Pyritization, Antennal scale, Tagmosis, Dorsoventral segmental mismatch, Computed tomography

## Abstract

**Background:**

Artiopodan euarthropods represent common and abundant faunal components in sites with exceptional preservation during the Cambrian. The Chengjiang biota in South China contains numerous taxa that are exclusively known from this deposit, and thus offer a unique perspective on euarthropod diversity during the early Cambrian. One such endemic taxon is the non-trilobite artiopodan *Sinoburius lunaris*, which has been known for approximately three decades, but few details of its anatomy are well understood due to its rarity within the Chengjiang, as well as technical limitations for the study of these fossils. Furthermore, the available material does not provide clear information on the ventral organization of this animal, obscuring our understanding of phylogenetically significant details such as the appendages.

**Results:**

We employed X-ray computed tomography to study the non-biomineralized morphology of *Sinoburius lunaris*. Due to the replacement of the delicate anatomy with pyrite typical of Chengjiang fossils, computed tomography reveals substantial details of the ventral anatomy of *Sinoburius lunaris*, and allow us to observe in detail the three-dimensionally preserved appendicular organization of this taxon for the first time. The dorsal exoskeleton consists of a crescent-shaped head shield with well-developed genal spines, a thorax with seven freely articulating tergites, and a fused pygidium with lateral and median spines. The head bears a pair of ventral stalked eyes that are accommodated by dorsal exoskeletal bulges, and an oval elongate ventral hypostome. The appendicular organization of the head is unique among Artiopoda. The deutocerebral antennae are reduced, consisting of only five podomeres, and bear an antennal scale on the second podomere that most likely represents an exite rather than a true ramus. The head includes four post-antennal biramous limb pairs. The first two biramous appendages are differentiated from the rest. The first appendage pair consists of a greatly reduced endopod coupled with a greatly elongated exopod with a potentially sensorial function. The second appendage pair carries a more conventionally sized endopod, but also has an enlarged exopod. The remaining biramous appendages are homonomous in their construction, but decrease in size towards the posterior end of the body. They consist of a basipodite with ridge-like crescentic endites, an endopod with seven podomeres and a terminal claw, and a lamellae-bearing exopod with a slender shaft. Contrary to previous reports, we confirm the presence of segmental mismatch in *Sinoburius lunaris*, expressed as diplotergites in the thorax. Maximum parsimony and Bayesian phylogenetic analyses support the monophyly of Xandarellida within Artiopoda, and illuminate the internal relationships within this enigmatic clade. Our results allow us to propose a transformation series explaining the origin of archetypical xandarellid characters, such as the evolution of eye slits in *Xandarella spectaculum* and *Phytophilaspis pergamena* as derivates from the anterolateral notches in the head shield observed in *Cindarella eucalla* and *Luohuilinella* species. In this context, *Sinoburius lunaris* is found to feature several derived characters within the group, such as the secondary loss of eye slits and a high degree of appendicular tagmosis. Contrary to previous findings, our analyses strongly support close affinities between *Sinoburius lunaris*, *Xandarella spectaculum* and *Phytophilaspis pergamena*, although the precise relationships between these taxa are sensitive to different methodologies.

**Conclusions:**

The revised morphology of *Sinoburius lunaris*, made possible through the use of computed tomography to resolve details of its three-dimensionally preserved appendicular anatomy, contributes towards an improved understanding of the morphology of this taxon and the evolution of Xandarellida more broadly. Our results indicate that *Sinoburius lunaris* possesses an unprecedented degree of appendicular tagmosis otherwise unknown within Artiopoda, with the implication that this iconic group of Palaeozoic euarthropods likely had a more complex ecology and functional morphology than previously considered. The application of computer tomographic techniques to the study of Chengjiang euarthropods holds exceptional promise for understanding the morphological diversity of these organisms, and also better reconstructing their phylogenetic relationships and evolutionary history.

## Background

The Artiopoda Hou and Bergström [1] comprise a major group of largely benthic marine euarthropods with a distinctively flattened appearance that thrived throughout most of the Palaeozoic Era, before reaching their demise in the Permian-Triassic mass extinction event. Although trilobites are by far the most speciose and familiar artiopodans by virtue of possessing a heavily calcified dorsal exoskeleton conducive to fossilization [2], the early evolutionary history of this diverse clade includes several non-biomineralized forms that are largely known from sites of exceptional preservation around the world [3–5]. The taxa represented in these Konservat-Lagerstätten provide unique insights into the morphology, ecology and evolutionary relationships of extinct euarthropods thanks to their superior preservation quality, which often include details of the ventral exoskeletal and appendicular organization. The Chengjiang biota in Yunnan Province, South China, represents one of the most influential Konservat-Lagerstätten in this regard, as its continuous study spanning several decades has produced a wealth of detailed anatomical information leading to a better understanding of the palaeobiology of early euarthropods [1, 6, 7]. Whereas the early Cambrian deposits in Yunnan Province have produced valuable morphological data that informs the evolution of euarthropods, including aspects of the head and trunk appendages [6, 8, 9], digestive tract [10–12], and even nervous systems [13–15], these exceptional fossils have been largely studied using traditional imaging techniques. Some recent developments include the application of fluorescence microscopy in order to increase the contrast between body fossils and the rock matrix, which has allowed to visualize minute features [15, 16]. However, the fundamental limitation of these methodologies is that they only capture surface information from any given fossil, and thus the morphological details preserved inside the rock matrix remain largely inaccessible in most specimens (notwithstanding burial orientation) given the difficulty of removing the surrounding sediment at such a fine scale. The recent implementation of X-ray based computed tomography to study Chengjiang fossils has opened the doors for new discoveries on the cryptic preservation of the proximal appendicular organization in early euarthropods, and already led to the discovery of phylogenetically significant features in problematic taxa [17–19]. Here, we describe in detail the appendicular morphology of the non-biomineralized euarthropod *Sinoburius lunaris* Hou, Ramsköld & Bergström, 1991 [20], one of the least understood Chengjiang artiopodans, by employing computer tomography to visualize the three-dimensional structure of the preserved appendages. The new data on the limb organization of *S. lunaris* lead to a better understanding of Xandarellida, and reveals a suite of significant morphological features that would otherwise be impossible to resolve with conventional photographic techniques alone.

## Results

### Systematic Palaeontology

Euarthropoda Lankester, 1904 [21] (see discussion in ref. [22]).

Artiopoda Hou & Bergström, 1997 [1].

Xandarellida Chen, Ramsköld, Edgecombe & Zhou in Chen et al., 1996 [23].

### Constituent taxa

*Cindarella eucalla* Chen, Ramsköld, Edgecombe & Zhou in Chen et al. 1996 [23], *Luohuilinella deletres* Hou, Williams, Sansom, Siveter, Siveter, Gabbott, Harvey, Cong & Liu 2018 [24], *Luohuilinella rarus* Zhang, Fu, & Dai, 2012 [25], *Phytophilaspis pergamena* Ivantsov, 1999 [26], *Sinoburius lunaris* Hou, Ramsköld & Bergström, 1991 [20], *Xandarella mauretanica* Ortega-Hernández et al., 2017 [27], *Xandarella spectaculum* Hou, Ramsköld & Bergström, 1991 [20].

### Emended diagnosis

Artiopodans with broad semicircular head shield with stalked lateral eyes originating ventrally. Eyes are accommodated in either anterolateral notches in the head shield, or dorsal exoskeletal bulges. Head shield covers uniramous antennae and four or more pairs of biramous appendages. Natant hypostome with elongate suboval outline, situated far behind anterior margin of head shield. Head shield extended posteriorly to cover multiple thoracic tergites. First thoracic tergite variably reduced. Thoracic tergites with variable patterns of dorsoventral mismatch relative to number of biramous appendage pairs. Endopods slender and with small or no endites. Fused pygidium variable in size. Pygidium with broad median spine in most forms. Modified from ref. [28].

### Remarks

The new data on *Sinoburius lunaris* leads us to propose a more accurate diagnosis for Xandarellida [23] that better reflects the morphology of its constituent taxa. This represents an update from the emended diagnosis provided more recently by Ortega-Hernández et al. [27], which took into consideration aspects of the ventral morphology based on Ramsköld et al. [28]. We regard the original diagnosis of the higher taxon Petalopleura Hou and Bergström, 1997 [1] as insufficiently specific given its lack of focus on characters that would effectively differentiate these euarthropods from other artiopodans. The phylogenetic analysis of Edgecombe and Ramsköld [6] supported Petalopleura based on the overlap of the anterior trunk tergites by the head shield, and the presence of an axial spine on the posterior trunk tergite. The revised morphology of *S. lunaris* indicates that an axial spine is in fact absent, and thus cannot be used to support this clade (see below). Given these observations, we consider that the utility of Petalopleura as a higher systematic unit is somewhat limited, and that its purpose is better embodied by Xandarellida as redefined here.

**Fig. 1:**
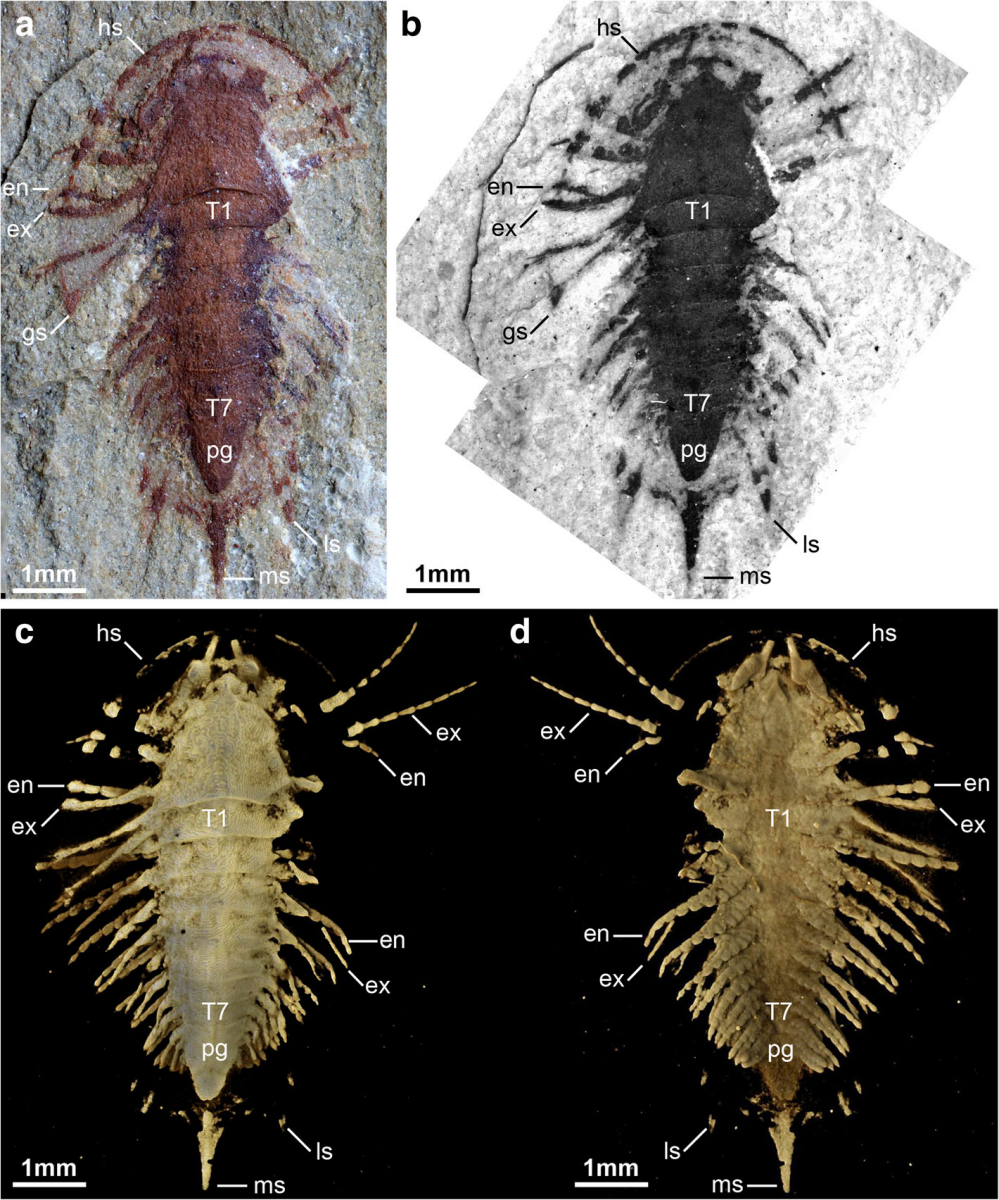
The non-biomineralized artiopodan *Sinoburius lunaris* from the early Cambrian (Stage 3) Chengjiang. **a** YKLP 11407, dorsal view of specimen photographed under light microscopy. **b** Dorsal view of specimen photographed under fluorescence microscopy. **c** Dorsal view of three-dimensional computer model based on X-ray tomographic data rendered in Drishti [73]. **d** Ventral view of three-dimensional model based on X-ray tomographic data. Abbreviations: en, endopod; ex, exopod; gs, genal spine; hs, head shield; ls, lateral spine; ms, median spine; pg, pygidium; T*n*, thoracic segment

**Fig. 2:**
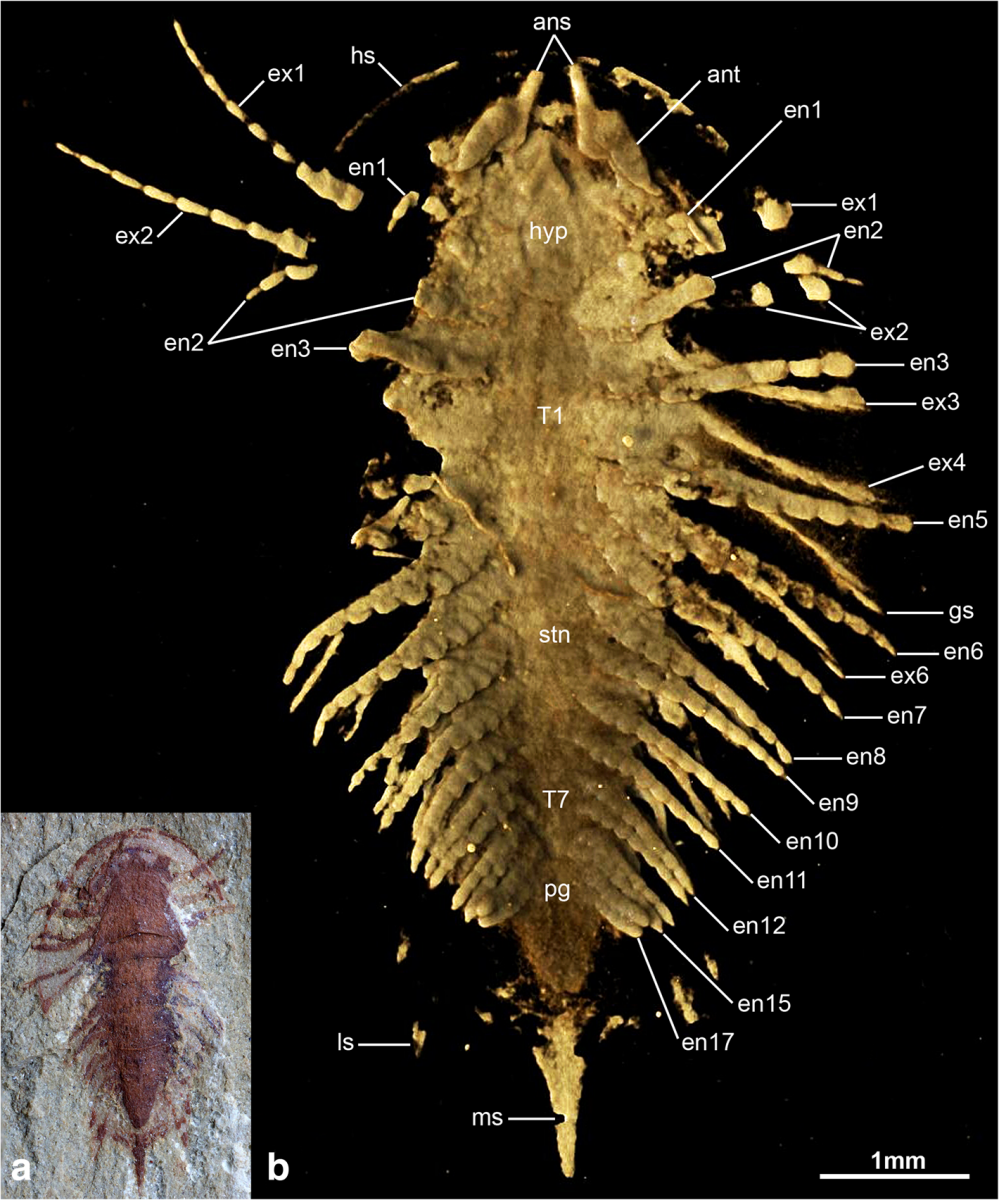
Detailed ventral morphology of *Sinoburius lunaris*, specimen YKLP 11407. **a** Specimen photographed under light microscopy. **b** Ventral view of three-dimensional computer model based on X-ray tomographic data rendered in Drishti [73] showing details of the well-preserved appendages concealed by the rock matrix. Abbreviations: ans, antennal scale; ant, antennae; en, endopod; ex, exopod; hs, head shield; hyp, hypostome; ls, lateral spine; ms, median spine; pg, pygidium; stn, sternite; tc, terminal claw; T*n*, thoracic segment

**Fig. 3:**
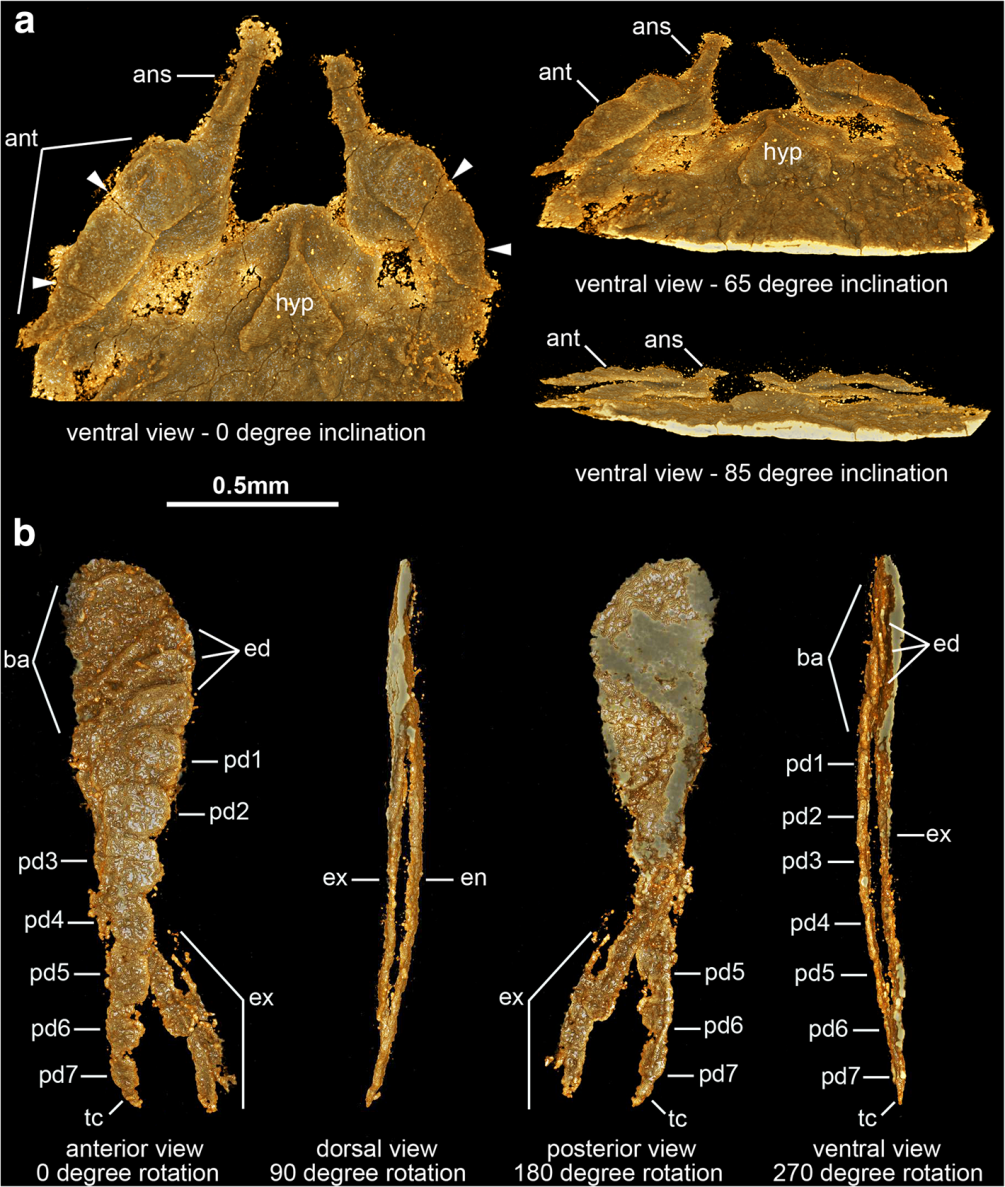
Cephalic and limb morphology of *Sinoburius lunaris*, specimen YKLP 11407. **a** Three-dimensional computer model of ventral view of anterior cephalic region based on X-ray tomographic data rendered in Drishti [73]. Arrowheads indicate podomere boundaries in antennae. **b** Three-dimensional computer model of virtually dissected ninth biramous appendage from right side of body. Abbreviations: ans, antennal scale; ant, antennae; ed., endite; en, endopod; ex, exopod; hyp, hypostome

The recent description of the genus *Luohuilinella* [24, 25], and the suggestion that *Xandarella* may be present in the middle Cambrian of Morocco [27], have increased the diversity of Xandarellida to a total of seven species. However, these taxa also embody a significant degree of morphological variability [29], as exemplified by the drastically divergent patterns of dorsal exoskeletal tagmosis observed between *Luohuilinella* and *Cindarella* relative to *Phytophilaspis* [26]. To further complicate matters, our observations demonstrate that some characters previously considered to broadly define the group, such as dorsal eye slits or an axial spine on the posterior part of the body [see characters 7 and 23 respectively in ref. [6], are expressed in fewer than half of these taxa. Instead, we find that more reliable indicators of affinities within Xandarellida include a particular combination of characters rather than specific autapomorphies. For example, the posterior articulation of the head shield with a reduced thoracic tergite appears to be synapomorphic and the only character observable in all representatives of Xandarellida, even if this feature is also found in taxa outside this group (e.g. *Zhiwenia coronata* [30], *Tremaglaspis vanroyi* [31]. The occurrence of dorsoventral segmental mismatch between tergites and biramous limb pairs is also expressed in all members of Xandarellida that feature both appendicular and exoskeletal preservation, namely *Cindarella, Sinoburius,* and *Xandarella* [1, 6, 28]. All xandarellids also share the presence of ventral stalked eyes, although they may also be accommodated dorsally in an exoskeletal bulge (see char. 6 in ref. [6]. Although *Phytophilaspis* has been regarded as having dorsal sessile eyes [26, 32], published photographs of the type material suggest that the eyes are not sharply defined within the head shield (see Fig. 2a-d in ref. [26]. Instead, the smooth transition between the convex ocular structures and the head shield of *Phytophilaspis* closely resembles the raised exoskeletal bulges that typify *Xandarella* and *Sinoburius*, and thus is suggestive of the ventral origin of the eyes (see discussion below). Finally, where the hypostome is known in xandarellids it is consistently natant, elongate, and situated a considerable distance away from the anterior margin of the head shield, as observed in *Cindarella* [28]*, Luohuilinella* [24], *Phytophilaspis* [26]*, Sinoburius* (Figs. 1, 2 and 3)*, Xandarella spectaculum* [7] and likely also *Xandarella mauretanica* [27].

*Sinoburius* Hou, Ramsköld & Bergström, 1991 [20].

#### Type species

*Sinoburius lunaris* Hou, Ramsköld & Bergström, 1991 [20].

### Emended diagnosis

Head shield large relative to total body length, crescentic in outline, with paired exoskeletal bulges accommodating ventral stalked eyes situated mediolaterally. Head shield covers a pair of antennae and four pairs of biramous appendages. Antennae short, and proximally bear an antennal scale. First and second biramous limbs with elongate stenopodous exopods, morphologically distinct from trunk exopods. First endopod pair greatly reduced. Thorax consisting of seven freely articulating tergites. First thoracic tergite with reduced pleurae and partly covered by head shield. Biramous appendages consist of gnathobasic basipodite, endopod with seven podomeres without endites, and setae-bearing exopod composed of slender shaft with small distal lobe. Segmental mismatch between thoracic tergites and number of biramous appendages. Pygidium with well-developed median spine, and two pairs of smaller lateral spines. Pygidium covers at least four appendage pairs and a conical tail piece. Modified from ref. [1].

### Remarks

*Sinoburius* is among the least studied Chengjiang artiopodans, which partly stems from the rarity of this taxon and its restricted stratigraphic occurrence compared to some of the more common components of this biota [1, 6, 20, 33], such as the nektaspidids [10]. Since its original description [20], the morphology of *Sinoburius* was slightly revised by Hou and Bergström [1] based on a few additional specimens, but no further details of the ventral anatomy were available given the preservation of the fossils. Likewise, Edgecombe and Ramsköld [6] addressed the dorsal morphology of this taxon based on newly figured specimens and discussed specific aspects of its exoskeletal organization in the context of Xandarellida, but were again limited by the material available. Here, we provide a more accurate diagnosis of *Sinoburius* based on fluorescence microscopy and new three-dimensionally preserved appendicular data obtained through X-ray based computer tomography [17–19].

*Sinoburius lunaris* Hou, Ramsköld & Bergström, 1991 [20].

1991 Hou, Ramsköld & Bergström, pp. 403, Fig. 4 [20].

1996 Chen et al., pp. 165, Figs. 214, 215 [23].

1997 Luo et al., pp. 102, pl. 2, Fig.4 [33].

1997 Hou & Bergström, pp. 83–86, Figs. 77–79 [1].

1999 Luo et al., pp. 50, 51, pl. 7, Fig. 1 [34].

1999 Edgecombe & Ramsköld, p. 264, Fig. 1 [6].

1999 Hou et al., pp. 125, Figs. 179–181 [35].

2003 Bergström & Hou, pp. 326, Fig. 3c [29].

2004 Hou et al., pp. 174, 175, Figs. 16.61, 16.62 [36].

2004 Chen, pp. 266, Figs. 414, 415 [37].

2017 Hou et al., pp. 202, 203, Figs. 20.32, 20.33 [7].

#### Diagnosis

As for genus.

### Description

**Fig. 4:**
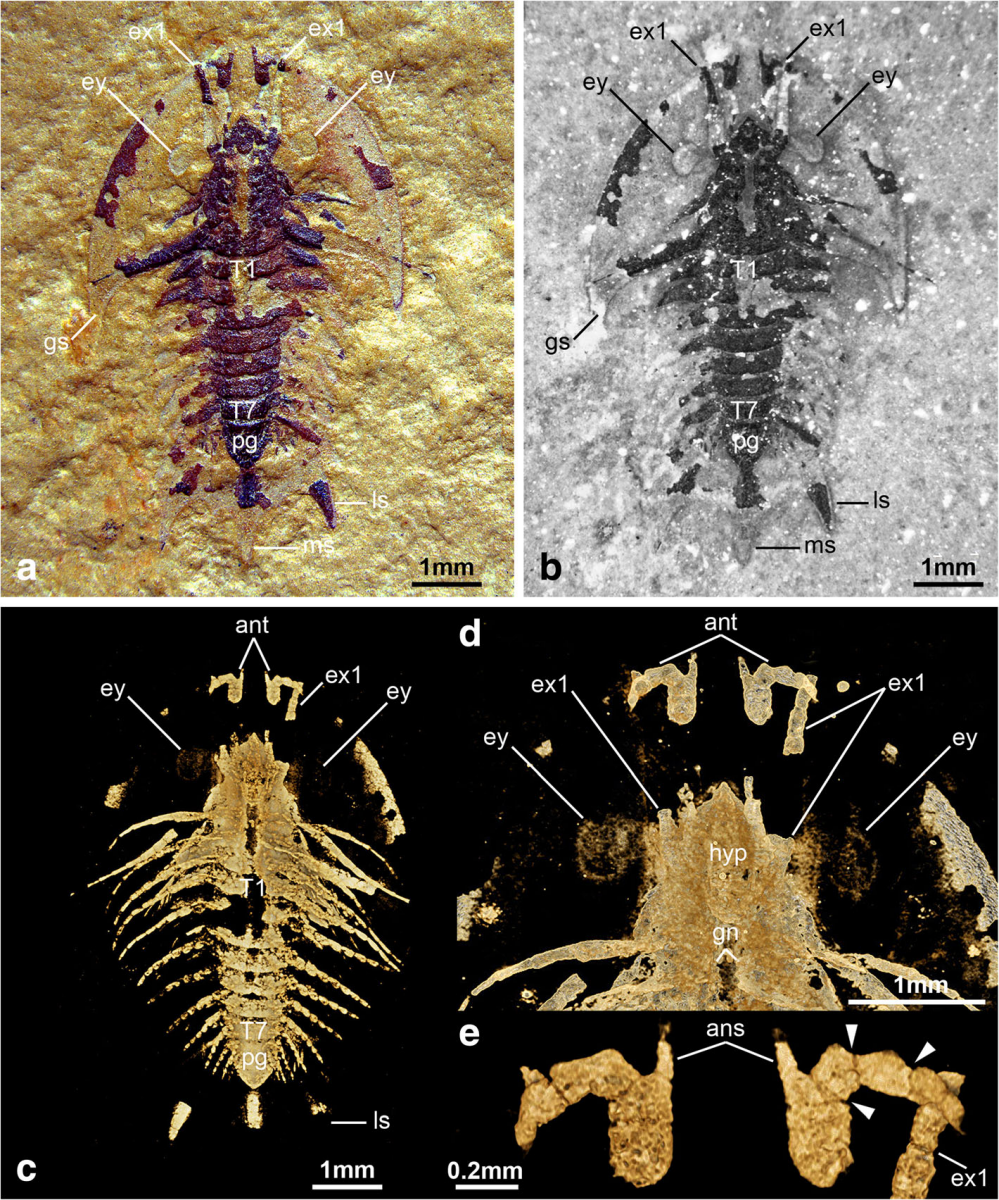
The non-biomineralized artiopodan *Sinoburius lunaris* from the early Cambrian (Stage 3) Chengjiang. **a** YRCP 0011, dorsal view of specimen photographed under light microscopy. **b** Dorsal view of specimen photographed under fluorescence microscopy. **c** Ventral view of three-dimensional computer model based on X-ray tomographic data rendered in Drishti [73]. **d** Close up of head region in ventral view. **e** Close-up of uniramous antennae with antennal scale. Arrowheads indicate podomere boundaries in antennae. Abbreviations: ans, antennal scale; ant, antennae; en, endopod; ex, exopod; ey, eye; gs, genal spine; gn, gnathobases; hs, head shield; hyp, hypostome; ls, lateral spine; ms, median spine; pg, pygidium; T*n*, thoracic segment

Completely articulated specimens range between 7.2 to 8.04 mm in length (sagittal), measured from the anterior margin of the cephalon to the tip of the median spine on the pygidium (Figs. 1, 2, 4, 6). The dorsal exoskeleton consists of three distinct tagmata, comprising a semicircular head shield, a thorax with freely articulating tergites, and a fused pygidium (Figs. 1, 4, 6). The head shield has a pronounced crescentic outline resulting from the semicircular anterior margin, coupled with the extensive lateral genal angles (Fig. 1a, b). The head shield (including the genal angles) is proportionately large compared to the trunk, representing approximately half of the entire body length (sag.), and is at least 1.5 times wider (transverse) than the trunk (Figs. 1, 4, 6). The genal angles are broadly triangular and terminate in a short posterior-facing genal spine (Figs. 1a, 4a, 6a). The tips of the genal spines reach posteriorly to the level of the third trunk tergite (Figs. 1a, b, 4a, b, 6a, b). The posterior cephalic margin is anteriorly reflexed on its median region, accentuating the crescentic shape of the head shield (Figs. 1, 4, 6). The head shield features a pair of ovoidal dorsal exoskeletal bulges (se char. 9 in ref. [6] situated slightly anterior relative to the midline (trans.) of the head (Figs. 4a, b, 6a, b), and that accommodate a pair of ventral stalked eyes (Figs. 4c, d, 6c). There is no evidence of ecdysial sutures or other dorsal structures. The axial region of the head shield is slightly elevated, corresponding to the bulk of the underlying body, but not developed into a morphologically discrete well-defined glabella with furrows or lobes. The elevated sagittal area narrows anteriorly into an acute termination that conveys a bullet-like appearance to the axial region (Figs. 1a, b, 4a, b, 6a, c).

**Fig. 5:**
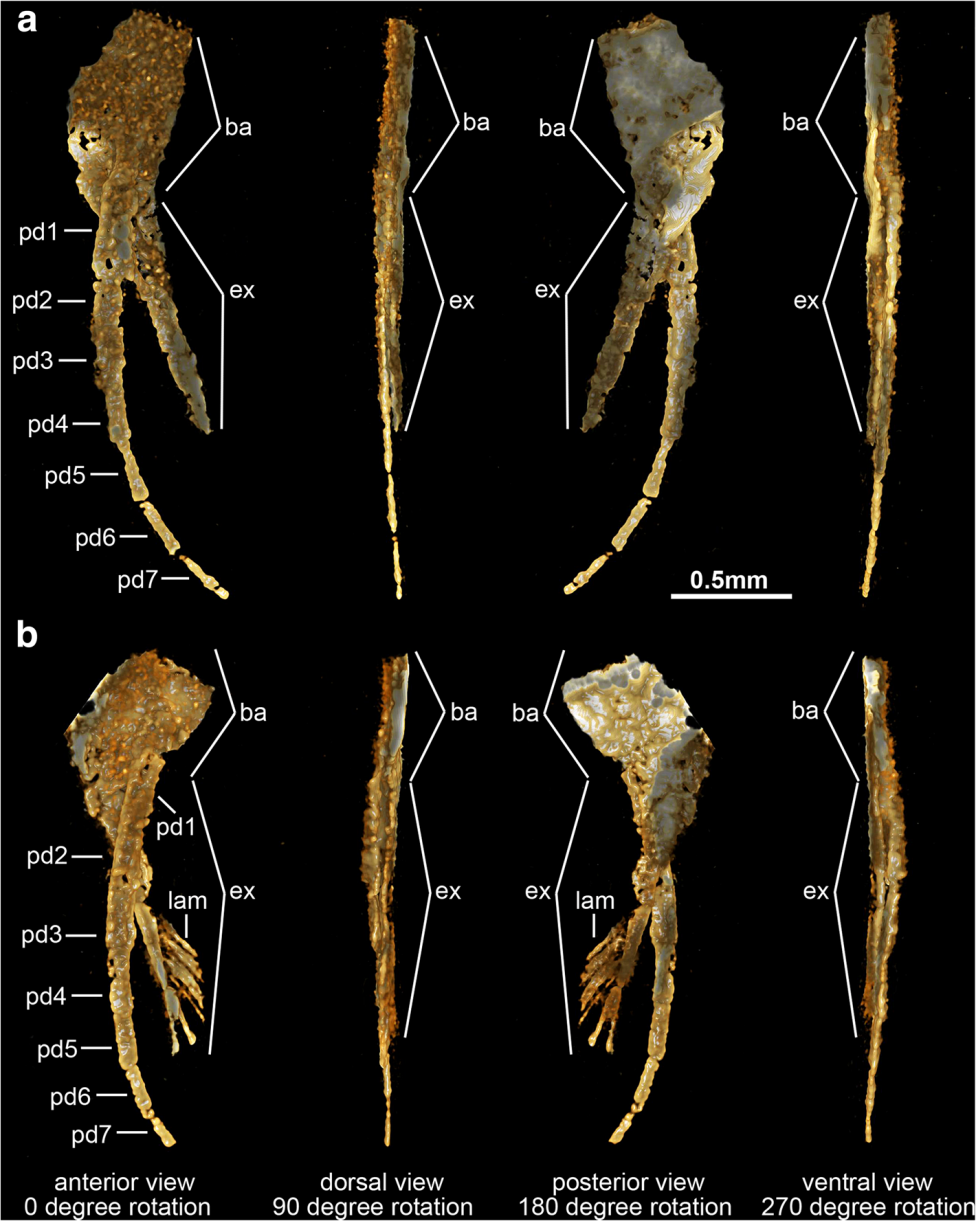
Morphology of biramous appendages in *Sinoburius lunaris* (YRCP 0011). **a** Third post-antennal limb pair. **b** Fourth post-antennal limb pair. Abbreviations: ba, basipodite; en, endopod; ex, exopod; lam, lamellae; pd*n*, podomere

**Fig. 6:**
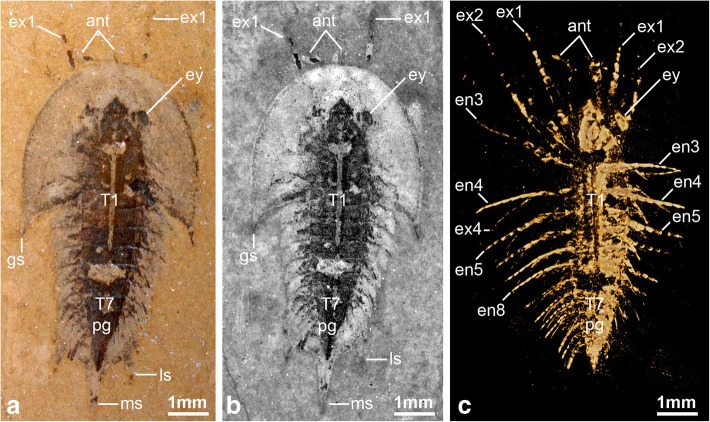
The non-biomineralized artiopodan *Sinoburius lunaris* from the early Cambrian (Stage 3) Chengjiang. **a** Hz-f-10-45, dorsal view of specimen photographed under light microscopy. **b** Dorsal view of specimen photographed under fluorescence microscopy. **c** Dorsal view of three-dimensional computer model based on X-ray tomographic data rendered in Drishti [73]. Abbreviations: ant, antennae; en, endopod; ex, exopod; ey, eye; gs, genal spine; ls, lateral spine; ms, median spine; pg, pygidium; T*n*, thoracic segment

The ventral side of the head shield features a thin doublure along the margins, and an elongate natant hypostome with an overall ovoidal shape (Figs. 2b, 3a, b, 4d). The hypostome occupies approximately one third of the underside of the head, and is separated from both margins of the head by a similar third both anteriorly and posteriorly (Fig. 2b). Most of the hypostome surface consists of a flattened plate with an elongate teardrop-like appearance with an elevated lateral margin rim. The marginal rim of the hypostome is further developed on the anterior tip, with both sides converging into an abruptly elevated triangular structure resembling an inverted furcula (Figs. 2b, 3a). The cephalic appendages include a pair of antennae, followed by four pairs of biramous limbs (Figs. 1c, d, 2b). The antennae originate close to the anterior margin of the hypostome, as typically observed in the deutocerebral first limb pair observed in various Cambrian euarthropods [38]. The antennae are short, and consist of five podomeres that decrease in size distally as indicated by the presence of constrictions along their length and the presence of regular transverse breakages (Figs. 3a, 4d, e). The antennae do not face forwards as typically observed in artiopodans [1, 6, 10, 24, 27, 30, 39], but rather are splayed at either side due to the strong lateral flexure of the third to fifth podomeres. We regard the preserved orientation of the antennae in YRCP 0011 as close to life position (Fig. 4d, e), whereas the antennae in YKLP 11407 appear to be bent backwards due to compression during burial as evidenced by the gap between the antennae and the body (Fig. 3a). The basal podomere is robust and cup-shaped, whereas the more distal podomeres become progressively smaller in overall size until terminating in an acute tip (Fig. 4e). In both YKLP 11407 and YRCP 0011, each short antenna is associated with an anterior-facing straight spine-like structure that originates from the second podomere (Figs. 3a, 4d, e). The spine-like structure is slightly longer than the third antennal podomere. The distal tip of the spine-like structure is located close to the anterior margin of the head shield, but does not project beyond it (Figs. 2b, 4c, d). The first pair of biramous post-antennal appendages occupy a para-oral position, and are located at either side of the hypostome (Fig. 2b), in accordance with a tritocerebral segmental origin [38]. The endopod in the first limb pair is greatly reduced in size, and consists of only five podomeres without endites (Fig. 2b). In contrast, the corresponding exopod is elongate and stenopodous; it consists of at least a dozen well-defined podomeres that taper in size distally and lack any indication of preserved setae or lamellae. At least the distal half of the exopod extends beyond the margins of the head shield (Figs. 1c, d, 2b, 6). The second pair of biramous limbs occupies a post-oral position. The endopod has a more conventional construction and size. Although it is incompletely preserved, its size suggests that it consists of approximately seven podomeres without endites and a terminal claw. The preservation of the distal podomeres indicate that the second endopod is mostly covered by the head shield, with only the terminal claw extending beyond the margins of the dorsal exoskeleton in some specimens (Fig. 2b). The corresponding exopod is similar to that of the first biramous limb pair, namely it is elongate, stenopodous, and extends far beyond the head shield margins (Figs. 1c, d, 2b). Like the first pair, the second biramous limbs does not preserve evidence of setae or lamellae on the exopods. The third and fourth biramous appendage pairs in the head are similar to each other (Fig. 5). The endopods consist of approximately seven podomeres without endites, whose terminal claws extend close to the edge of the head shield (Fig. 4c). The exopods are considerably shorter compared to the previous appendages, and resemble the corresponding structures on the trunk appendages (see description below). The basipodite is visible in the third and fourth post-antennal limb pairs of YRCP 0011, and demonstrates the presence of faint gnathobasic endites that face adaxially (Figs. 4c, d, 5).

The thorax consists of seven freely articulating tergites that overlap widely with each other (Figs. 1, 4, 6). The thorax is approximately as long (sag.) as the distance between the posterior and anterior margins of the head shield. All the tergites are approximately of subequal length (sag.), and possess well-developed pleurae that curve into posterior-facing spines. The first thoracic tergite has reduced pleural regions and is the narrowest (trans.) point of the body; this tergite is also partly concealed by the posterior margin of the head shield in life position. The thorax is widest at the level of the third or fourth tergite depending on the individual, and subtly narrows towards the pygidium. The central portion of the tergites, corresponding approximately to a third of the tergite width (trans.), is slightly raised into a weak axial lobe that continues into the corresponding region of the cephalic shield. The fused pygidium is shorter (sag.) than the thorax. The pygidium bears two pairs of broad lateral spines directed backwards, and its posterior portion consists of a broad median spine with an acute termination (Figs. 1, 4, 6). The length (sag.) of the posterior median spine varies between individuals, and can be either shorter (Fig. 4) or subequal (Figs. 1, 6) relative to the main body of the pygidium (measured without the posterior spine).

The thorax and pygidium bear a series of homonomous biramous appendages that become smaller towards the posterior end of the body (Figs. 1d, 2b, 4c, 6c). Proximally, the biramous appendages possess a differentiated basipodite that bears three ridge-like crescentic endites (Figs. 3b, 4). In the thorax, the endopods consist of seven podomeres without endites, and a single terminal claw. There is some variability in the robustness of the endopods throughout the body, with those near the anterior region being more gracile (Fig. 4) compared to the ones towards the middle portion of the thorax (Figs. 2b, 3b). The exopod is composed of a slender shaft consisting of two or three elongate podomeres, and terminated on a small rounded distal lobe. Both the slender shaft and the distal lobe bear delicate lamellae (Figs. 1b, 2b, 3b, 5), although these are best preserved on the distal portions of the exopod (Figs. 1b, 5b). Most of the trunk tergites are associated with a single pair of biramous limbs. However, segmental mismatch is expressed in the presence of diplotergites (i.e. two appendage pairs associated with one tergite, see ref. 40, 41) that appear to be variably situated along the body. In YKLP 11407, diplotergites are expressed on the fourth and seventh thoracic tergites, whereas all the other tergites feature a single appendage pair (Figs. 1, 2). By contrast, both Hz-f-10-45 (Fig. 6) and YRCP 0011 (Fig. 4) have a diplotergite on the seventh trunk tergite only, although these taxa differ in the presence of four or three biramous limb pairs under the pygidium respectively. The size and podomere number of the endopods decreases gradually towards the posterior end, with some of the posteriormost endopods featuring only five observable podomeres (Fig. 2b). The ventral side of the body shows regular changes in texture that suggest the presence of sternites, but these are not well preserved (Fig. 2b). The pygidium covers at least four pairs of biramous appendages. The reduced podomere count and small size of the endopods under the pygidium resemble limb-buds rather than fully developed biramous appendages. The posterior body termination consists of a conical tail piece without limbs (Figs. 2b, 6c).

## Discussion

**Fig. 7:**
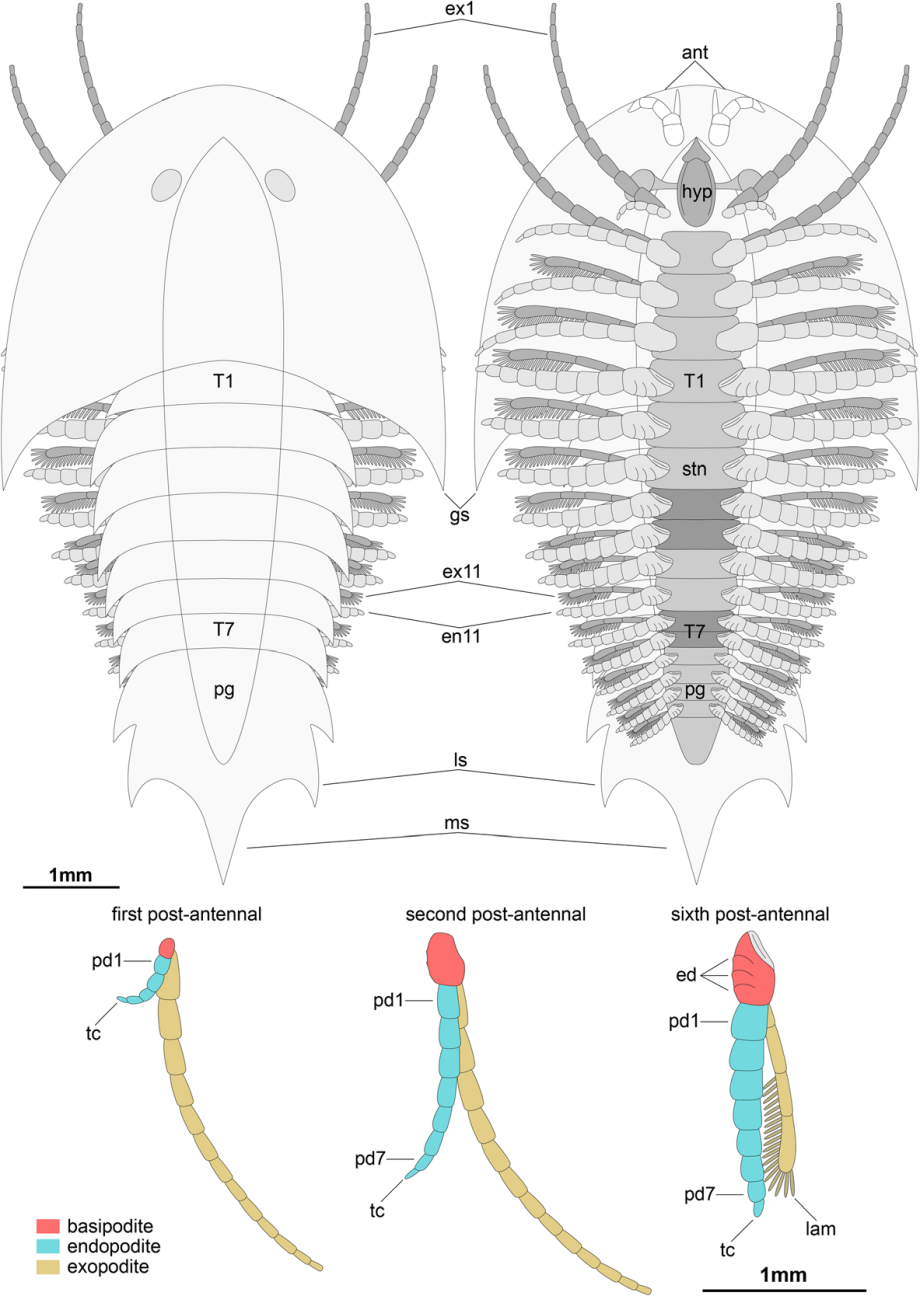
Morphological reconstruction of *Sinoburius lunaris* based on specimen YKLP 11407. Sternites associated with diplotergites highlighted. Abbreviations: ans, antennal scale; ant, antennae; ed., endite; en, endopod; ex, exopod; ey, eye; gs, genal spine; hyp, hypostome; lam, lamellae; ls, lateral spine; ms, median spine; pd*n,* podomere; pg, pygidium; stn, sternite; tc, terminal claw; T*n*, thoracic segment

The use of fluorescence microscopy and X-ray computed tomography reveal new details on the exoskeletal and appendicular morphology of *Sinoburius lunaris*, and allow us to provide a comprehensive redescription of this enigmatic taxon (Fig. 7). Of particular significance is the recovery of three dimensionally preserved anatomical information on the ventral side of the body (see also ref. [17–19]) that would be otherwise concealed by the surrounding rock matrix, made possible by the pyritization of Chengjiang soft-bodied fossils [42]. Our observations also allow a more detailed comparison with the preserved morphology of other xandarellids and more broadly within artiopodan diversity.

### Ecdysial sutures, lateral notches and eye slits

*Sinoburius lunaris* resembles most non-trilobite artiopodans in the absence of dorsal ecdysial sutures. However, some accounts of the morphology of this taxon have suggested the presence of eye slits (see char. 7 in ref. [6]; see also [32]), similar to those expressed in *Xandarella spectaculum* [1] and *Phytophilaspis pergamena* [26, 29, 32]. Edgecombe and Ramsköld [6] discussed a specimen figured by Hou and Bergström (see CN 115288 Fig. 77c, d in ref. [1]) as support for the presence of eye slits in *Sinoburius lunaris*. However, reexamination of CN 115288 illustrated by Hou et al. (see Fig. 20.33b in ref. [7]) demonstrates that that the furrow in the exoskeleton produced by the putative eye slits can be followed into the surrounding sediment as a fine line of dark material. Considering the new morphological data demonstrating the first and second exopod pairs are elongate and extend substantially beyond the head shield margins (Figs. 1c, d, 2b), and in the absence of clear dorsal features in otherwise well-preserved material (Figs. 1, 4, 6), we argue that the structures in CN 115288 are best regarded as impressions of the tritocerebral exopod rather than eye slits.

Du et al. [30] recently addressed the evolution of ecdysial sutures in Artiopoda based on the perceived similarity between the lateral notches in the head shield of *Zhiwenia coronata* and *Luohuilinella rarus* [25]. The preservation of the type material of *Luohuilinella rarus* gives the impression that this taxon may have possessed a similar cephalic organization to that of *Xandarella spectaculum* [1], including the possible presence of eye slits and dorsal exoskeletal bulges. The recent description of *Luohuilinella deletres* [24] shows that this is actually not the case. Instead, the head shield of *Luohuilinella* features deep lateral notches that accommodate the ventral stalked eyes, making it more comparable with the gentle notches of *Cindarella eucalla* [28], as well as the more profound lateral notches of non-xandarellid taxa such as *Zhiwenia coronata* [30] and *Sidneyia inexpectans* [43].

**Fig. 8:**
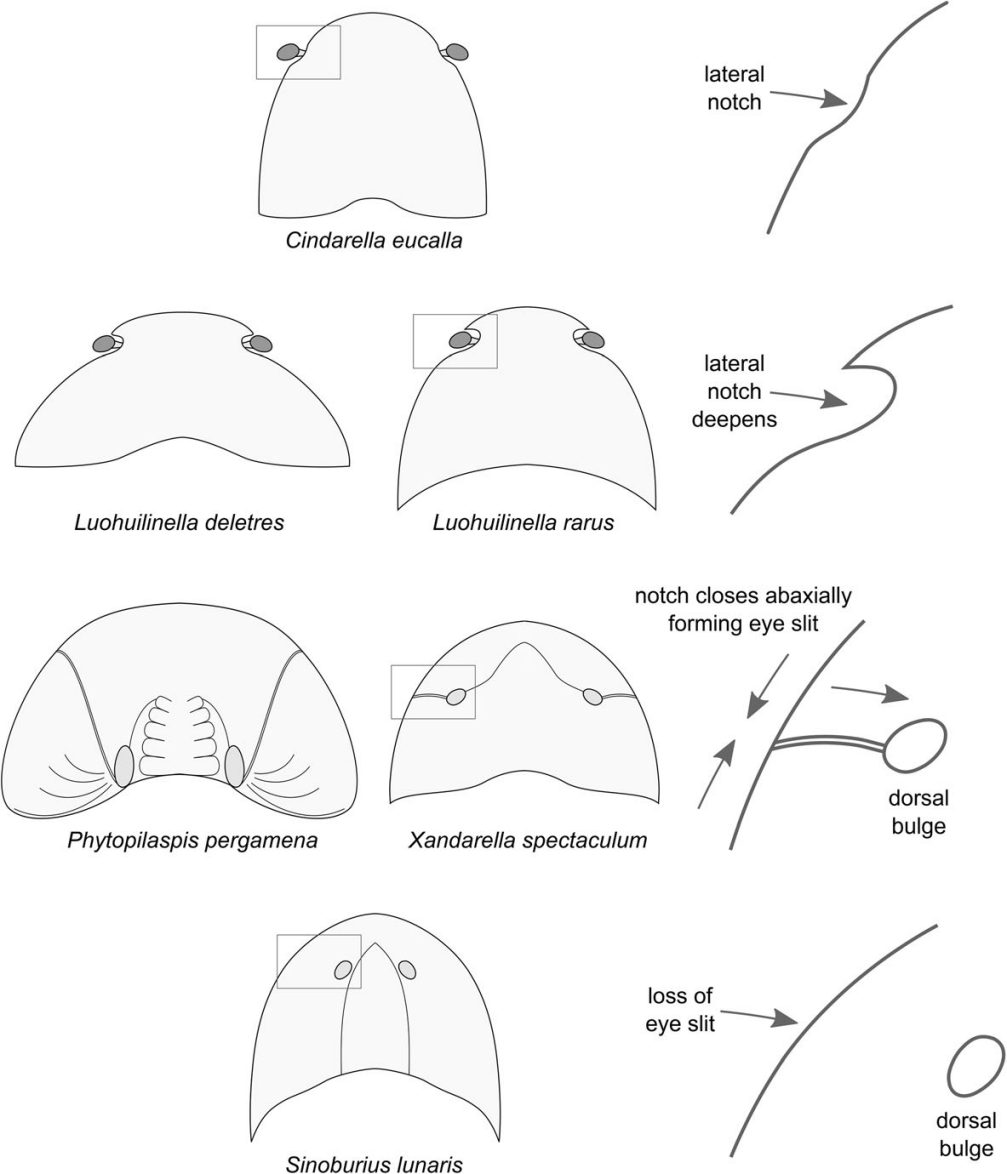
Evolution of anterolateral notches, eye slits and dorsal exoskeletal bulges in the head shield of Xandarellida. *Xandarella mauretanica* [27] is excluded since the dorsal exoskeleton is unknown

The improved understanding of xandarellid morphology conveyed by *Luohuilinella deletres* and the present revision of *Sinoburius lunaris* leads us to hypothesize an evolutionary transformation series resulting in the morphological diversity observed in Xandarellida. Given their common position on the anterolateral margins of the head shield, the notches expressed in *Cindarella* and *Luohuilinella* may reflect a progressive involution of the perforations that accommodate the ventral stalked eyes into the head shield (Fig. 8). This condition would be further developed in *Xandarella* and *Phytophilaspis*, in which the lateral notch has completely closed along the abaxial edges of the head shield, resulting in the eye slits in these taxa (Fig. 8). The absence of distinguishable lateral notches and eye slits in *Sinoburius* may indicate a more derived condition within the group. Alternatively, it is possible that eye slits may be expressed in this taxon, but given the small size of available specimens these structures could be too faint to be preserved in the fossils. Finally, we acknowledge that Zhang et al. [25] have previously suggested the homology between the lateral notches of *Luohuilinella* and the eye slits of *Xandarella*. Although we concur with the main sentiment expressed by Zhang et al. [25], it is necessary to clarify that the eye slits are not actually homologous with the notches in a strict sense, but rather that the eye slit form as a result of the adaxial integration of the lateral notch into the head shield (Fig. 8).

### Ventral eyes and exoskeletal bulges

Our observations confirm that *Sinoburius lunaris* possesses ventral stalked eyes (Fig. 4c, d), and that these are accommodated in dorsal exoskeletal bulges on the head shield (Fig. 4a, b) as suggested in previous studies [6]. In this context, revision of published material of *Xandarella spectaculum* (see Fig. 68–70 in ref. [1]) and *Phytophilaspis pergamena* (see Fig. 2a-d in ref. [26]) leads us to consider that these taxa also possess dorsal exoskeletal bulges that accommodate ventral eyes. For instance, it is well established that the eyes in *Xandarella* have a ventral origin and that the ocular structures on the head shield are perforations of the exoskeleton, rather than comparable with the dorsal eyes of other artiopodans such as trilobites [6, 28, 44]. However, the dorsal structures in *Xandarella* have not been explicitly interpreted as dorsal exoskeletal bulges in previous analyses (or at least scored as such) despite their morphological similarity with those of *Sinoburius* and some concilitergans (see ref. [5, 6, 32]), and particularly given the close phylogenetic relationships between *Xandarella* and *Sinoburius* themselves. Likewise, *Phytophilaspis* has been interpreted as featuring dorsal eyes [26], even though more recent accounts of its morphology favour the presence of eye slits similar to those of *Xandarella* [29, 32]. Given that the eye slits are a distinctive character that is only well documented in *Xandarella*, we argue that their presence in *Phytophilaspis* is likely also indicative of ventral eyes in this taxon. Furthermore, the putative dorsal eyes of *Phytophilaspis* lack a clear outline defining it from the rest of the cuticle on the head shield, but instead these features appear like elevations of the exoskeleton rather than discrete ocular structures. Based on these observations we tentatively consider that *Phytophilaspis* may also possess dorsal exoskeletal bulges accommodating ventral eyes similar to those in *Xandarella*.

The precise function of the eye slits in *Xandarella* and *Phytophilaspis* remains somewhat contentious, partly owing to the uniqueness of these features among Cambrian euarthropods. Hou and Bergström [1]; see also ref. [29] suggested that the notches, eye slits and dorsal exoskeletal bulges may have been involved in the capture of the ventral stalked eyes into the dorsal side of the head shield. Edgecombe and Ramsköld [6] acknowledged the novelty of the latter hypothesis, but concluded that it is not well supported. Our observations suggest that the notches, eye slits and dorsal bulges may indeed be linked to each other within a character transformation series among members of Xandarellida (Fig. 8), but cannot be extrapolated to the origin of dorsal eyes in other euarthropod groups.

### Natant hypostome

The xandarellid hypostome consists of an elongate plate that is situated medially (sag.) on the ventral side of the head, well separated from the anterior margin of the head shield (Figs. 2b, 3a), and shares a similar organization in all representatives where it has been observed [1, 24, 26–28]. The xandarellid natant hypostome deviates from that of most artiopodans, where generally consists of a concomitant plate that is in direct contact with the anterior margin of the head shield and occasionally bears an associated suture. Some examples of the concomitant organization include members of Aglaspididae [5, 45], *Retifacies abnormalis* [1], xenopods [39, 46] and *Kwanyinaspis maotiashanensis* [47]. The natant hypostome also differs from that of members of Conciliterga, in which this plate articulates with a semicircular anterior sclerite [1, 6, 48]. Nektaspidids represent the only other major group of trilobitomorphs that possess a natant hypostome [1, 6, 10, 32], although the xandarellid hypostome differs in having a distinctive elongate oval outline that is not present in former group.

### Short antennae with secondary branch

In most artipodan taxa the deutocerebral appendage is represented by a pair of uniramous antennae with a flagelliform organization, notwithstanding variation in the number of podomeres as well as their robustness and length [1, 10, 24, 28, 30, 46, 49]. The deutocerebral antennae of *Sinoburius lunaris* differ from those of all other known artiopodans in key aspects of this basic organization. The antennae are noticeably short and composed of only five podomeres (Figs. 3a, 4c–e), whereas the antennae of most other artiopodans will at least bear a dozen or so articles, up to a staggering 80 or more as exemplified by *Emeraldella brocki* [46]. Most notably, the deutocerebral appendage of *Sinoburius* bears an articulated structure similar to the ‘antennal scale’ or ‘outer scale’ observed on the antennule of extant leptostracan and bathynellacean crustaceans [50, 51], figured by Olesen (see Fig. 33.2B, C in ref. [52]). Among Cambrian artiopodans, the position of the antennal scale of *Sinoburius* resembles the so-called ‘ventral ramus’ described in *Misszhouia longicaudata* (see Fig. 35b in ref. [10]). In this case, the antennal scale of *Sinoburius* differs from that of *Misszhouia* in that the former is spine-like (Figs. 3a, 4e), whereas the latter appears to have distinct annulations. The antennal scale gives the appearance that the deutocerebral appendage has two rami, and has previously been considered as evidence of biramy in crustacean antennules [53, 54].

Despite the current naming scheme of the antennal scale in the literature, its implications for appendage morphology and development are somewhat misleading. In *Sinoburius lunaris*, *Misszhouia longicaudata*, and leptostracans, the scale is borne on the deutocerebral appendage. By contrast, a superficially similar structure (also called antennal scale, or sometimes ‘scaphocerite’) is borne on the tritocerebral appendage of caridoid crustaceans (i.e. all eumalacostracans with the exception of stomatopods) and some fossil ‘phyllocarids’ [54–58]. The tritocerebral antennal scales represent an unsegmented derivative of the antennal exopod, and therefore correspond to a true biramous limb (see Fig. 11.2 in ref. [58]). Deutocerebral appendages are uniramous in all known euarthropods [50, 51]. In extant leptostracans, the deutocerebral antennal scale is developmentally derived from the accessory flagellum, a secondary distal limb axis that lacks intrinsic musculature [51] and thus is an exite (sensu ref. [57]) rather than a true exopod [59]. In the absence of developmental information, we propose that the antennal scales of *Sinoburius lunaris* and *Misszhouia longicaudata* are analogous to those of leptostracans, namely they represent exites as part of the uniramous deutocerebral appendages, rather than legitimate rami.

### Reduced first endopod

The endopod of the first biramous appendage pair in *Sinoburius lunaris* is noticeably small compared to the remaining biramous appendage in the body, further emphasized by the fact that its corresponding exopod is substantially enlarged (Fig. 2b). It is not uncommon that the first post-oral limb pair of artiopodans is smaller given its close proximity to the hypostome and posterior-facing mouth (e.g. *Emeraldella brockii* [46], *Campanamuta mantonae* [60]). However, the difference in size expressed in *Sinoburius* is noteworthy, particularly when considering that *Xandarella mauretanica* [27] shares a similar organization in that the second endopod is at least twice as long as the first. The endopod structure of *Sinoburius* strengthens the suggested xandarellid affinity of *Xandarella mauretanica*, which is otherwise only known from a detailed impression of the ventral appendages and hypostome, but lacks information of the dorsal exoskeleton.

### Exopod differentiation

Possibly the most surprising aspect of the ventral appendicular morphology of *Sinoburius lunaris* is the fact that the exopods of the first two pairs of post-oral biramous appendages are drastically differentiated in terms of their morphology, and likely also their function (Figs. 2b, 7). Although a small degree of limb differentiation is known for some artiopodans, such as the complete absence of the exopod (e.g. *Emeraldella brockii* [46], *Cheloniellon calmani* [61], the condition of *Sinoburius* is unique within the known appendage diversity of this clade. It is noteworthy that the elongate exopod morphology of the first and second post-oral biramous appendages somewhat resembles flagelliform antennae, particularly considering that the deutocerebral antennae of *Sinoburius lunaris* are much shorter than those observed in other artiopodans (Figs. 3a, 4d, e). This juxtaposition of derived appendage morphologies suggests that the anterior exopods most likely substituted the antennae in terms of their sensorial tactile function as commonly observed in artiopodans. This scenario seems to be further supported by the fact that most of the post-oral limbs have lamellae-bearing exopods, arguably with a more conventional functional morphology for either respiration or water ventilation [62]. The co-option of post-deutocerebral limb pairs for a sensorial function is known in extant taxa, with analogues to the anterior exopods of *Sinoburius* found in the flagelliform post-tritocerebral limb pair of amblypygids (whip spiders), or the post-tritocerebral limb pair with elongate tarsi of uropygids (vinegaroons). A more apt comparison can be drawn with isopod crustaceans. For example, the embryonic development of the oniscid *Porcellio scaber* demonstrates that the deutocerebral antenna in the adult is greatly reduced, whereas the tritocerebral appendages have a more conventional antenniform morphology in line with a sensorial function [63], although in this case the endopod is enlarged, rather than the exopod as in *Sinoburius.* Branchiopod crustaceans also feature a small pair of deutocerebral appendages, themselves associated with a reduction of the corresponding brain neuromere, and extremely well-developed tritocerebral appendages [64].

### Gnathobasic basipodite

The flattened dorsal exoskeleton of artiopodans frequently obscures the proximal region of the body, particularly that of the attachment site between the body wall and the appendages. Computed tomography allows us to examine the proximal organization of the biramous appendages of *Sinoburius lunaris* in unprecedented detail, revealing the presence of crescent-shaped endites on the basipodite, and the precise attachment of the endopod and exopod to the former structure (Figs. 3b, 5, 7). The morphology of the basipodite is unknown for most members of Xandarellida with preserved appendages [1, 24, 28], although the presence of gnathobasic limbs has been suggested for *Xandarella spectaculum* in various phylogenetic analyses based on the possession of endopods with spinose endites distally [6, 32, 60]. The proximal morphology of *Sinoburius lunaris* demonstrates that it bore endites on the basipodite, most likely with a feeding function as observed in other Cambrian euarthropods [65–67]; however, the endites of *Sinoburius* differ from those of other forms in that they have a ridge-like crescentic outline, as opposed to a more typical spinose shape. Since *Sinoburius* endites are best resolved from a highly magnified close up of the computed tomography model (Fig. 3b), it is unlikely that these delicate structures would be clearly observable in body fossils under normal lighting conditions. Thus, it remains possible that other xandarellids may also possess endites with a similar morphology and feeding function, particularly since *Cindarella* and *Xandarella* possess well-developed mid-gut glands [1, 7, 28], which suggest complex food processing capabilities [11, 12].

### Reduced first thoracic tergite

**Fig. 9:**
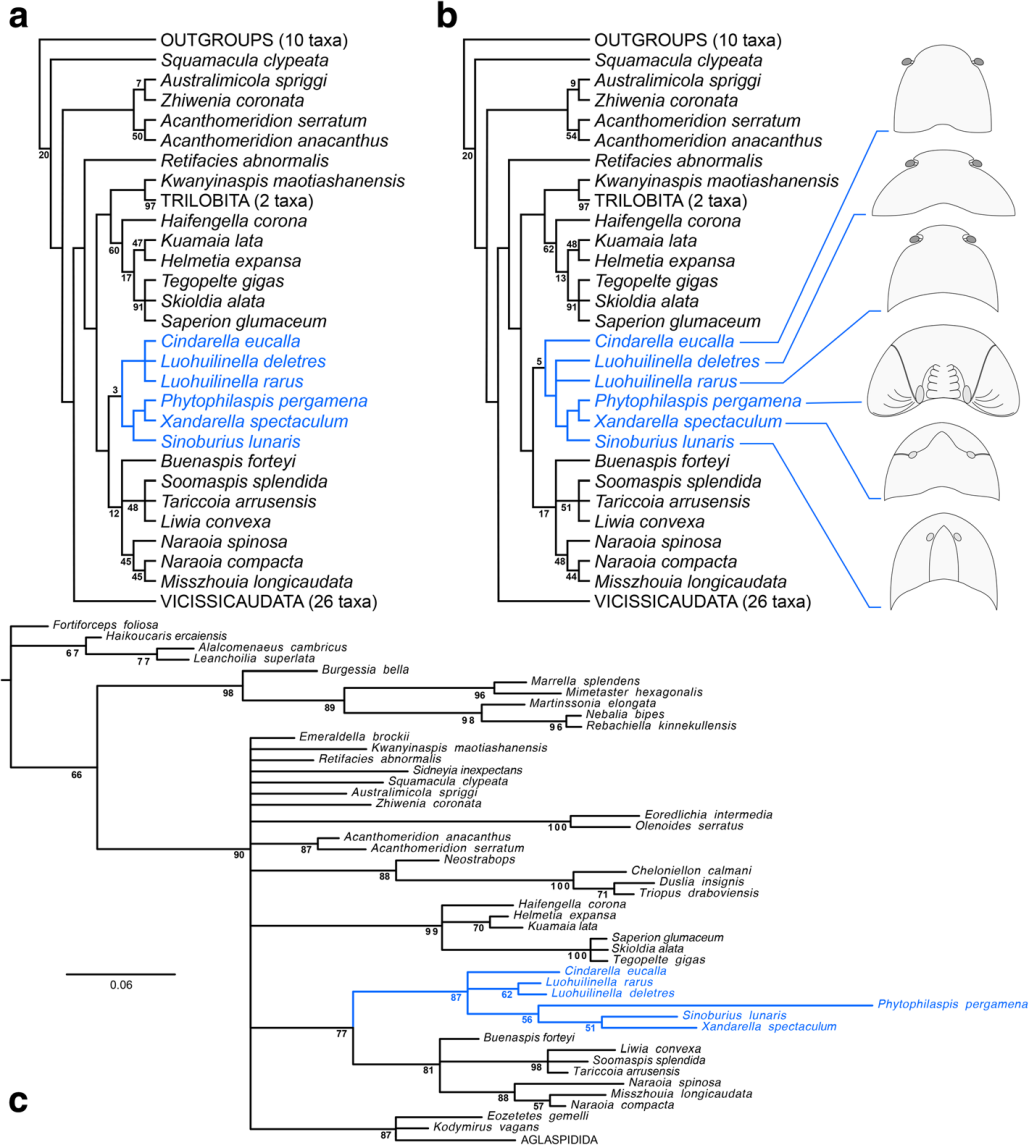
Phylogeny of Xandarellida (highlighted) within Artiopoda. **a** Maximum parsimony with implied weights (*k* = 4), strict consensus of 2 most parsimonious trees (CI = 0.412, RI = 0.737); node support values expressed as Group present/Contradicted frequency differences. **b** Maximum parsimony with implied weights (*k* = 5), strict consensus of 2 most parsimonious trees (CI = 0.413, RI = 0.739); node support values expressed as Group present/Contradicted frequency differences. **c** Consensus tree resulting from Bayesian analysis in MrBayes. Mk model, four chains, 1000,000 generations, 1/1000 sampling resulting in 5000 samples, 25% burn-in resulting in 3750 samples retained. Numbering denotes node posterior probability values. Internal topologies of Vicissicaudata and Aglaspidida same as those reported by Du et al. [30]

Xandarellida represents one of the most disparate groups within Artiopoda due to their divergent patterns of exoskeletal tagmosis, including forms with freely articulating tergites entirely (e.g. *Cindarella*, *Luohuilinella*), variably fused posterior tergites (*Xandarella*), and even an almost entirely ankylosed dorsum (*Phytophilaspis*) [1, 24, 25, 28, 29]. It is then not surprising that there are few exoskeletal characters that unite the group and distinguish them from other artiopodans. Although the evolution of the lateral notches and eye slits seems to be unique to xandarellids, the dynamics of this transformation series only allow us to identify their significance in a phylogenetic context (Figs. 8, 9). The presence of a reduced first trunk tergite is one of the best indicators of xandarellid affinities, as this character is expressed in all described representatives, the only exception being *Xandarella mauretanica* as the dorsal exoskeleton is yet unknown [27]. In *Sinoburius lunaris*, the pleural region of the first tergite is narrower (trans.) compared to the rest of the thorax (Fig. 4a, b), and similarly to other xandarellids it is substantially covered by the head shield (Fig. 6a, b). The reduction of the first tergite is restricted to the pleurae, however, as the axial region is similar to that of the second tergite both in terms of width (trans.) and length (sag.).

Xandarellids are the only trilobitomorphs with a reduced first trunk tergite. However, this character is also present in the artiopodans *Zhiwenia coronata* [30] and *Tremaglaspis vanroyi* [31], as well as other more distantly related taxa such as total-group euchelicerates [68]. Given the otherwise divergent aspects of the exoskeletal organization between all these taxa, the presence of a reduced first tergite among xandarellid and non-xandarellid taxa appears to be best regarded as a result of convergence, even if the functional or selective value of this adaptation remains somewhat enigmatic.

### Dorsoventral segmental mismatch

One of the distinguishing features of xandarellids with preserved limbs is the presence of dorsoventral segmental mismatch between the trunk tergites and the number of appendage pairs associated with them [6, 7, 28]. Segmental mismatch expressed as supernumerary limb pairs is relatively rare among Cambrian euarthropods [41], and only considered typical for certain groups in the Cambrian such as fuxianhuiids [1, 9, 12, 67] and euthycarcinoids [69]. Among artiopodans, this condition is usually expressed in the posterior body, for example the crowded appendages under the pygidium of the trilobite *Triarthrus eatoni* [70]. Both *Xandarella spectaculum* and *Cindarella eucalla* feature supernumerary limbs on the posterior half of the body, with the peculiarity that an increasing number of limb pairs are integrated into the more posterior tergites progressively [1, 28]. Although *Sinoburius lunaris* was previously thought to have a direct correspondence between the thoracic tergites and appendage pairs [6, 7], our data demonstrate that there is indeed segmental mismatch in this taxon expressed as diplotergites (i.e. two limb pairs per tergite [40]) in some parts of the thorax (Fig. 2b; Fig. 7), further consolidating this character as a defining feature of Xandarellida. It remains uncertain whether *Luohuilinella* also has supernumerary limbs as the type species does not preserve appendages [25], and the broad exoskeleton of *Luohuilinella deletres* does not allow to count the correlation between limbs and tergites posteriorly [24].

### Axial spine on posterior tergite

Previous studies that addressed the morphology of *Sinoburius lunaris* suggested the presence of an axial spine on a posterior trunk tergite (see char. 23 in ref. [6]). This observation was made based on a published photograph by Luo et al. (see plate 2, Fig. 4 in ref. [33]). Direct examination of figured specimen Hz-f-10-45 indicates that this feature is in fact absent (Fig. 6a, b). The low-angle illumination in the photograph published by Luo et al. [33] casts a strong shadow on the elevated sagittal ridge of the median spine in the pygidium of this specimen that gives the false impression of a distinct axial spine akin to that reported for *Cindarella eucalla* [6, 28]. Furthermore, we find no evidence of an axial spine in other studied materials despite its high quality of preservation. The presence of an axial spine has also been suggested for *Xandarella spectaculum* (see char. 23 in ref. [6]). However, we have not been able to corroborate the existence of this feature for *Xandarella*. In *Cindarella*, the posteriormost three tergites bear an elongate axial spine that originates from the sagittal region of dorsum [28], comparable to the axial spine observed in the thorax of *Eoredlichia intermedia* [71]. These particular axial spines of *Cindarella* differ from the broad median spine observed in *Sinoburius* and other taxa (see char. 65 in ref. [5]), in which the spine results from an extension of the posterior margin of the last tergite (Figs. 1a, b, 4a, b, 6a, b, 7). *Xandarella* clearly features a median spine similar to that of *Sinoburius*, but none of the specimens figured in the literature demonstrate the presence of an axial spine originating from the dorsal side of the posterior tergites that would be similar to those of *Cindarella* [1, 7]. We regard the presence of an axial spine as absent in *Xandarella*, and unique to *Cindarella* within the context of Xandarellida.

### Phylogenetic relationships within Xandarellida

The results of the phylogenetic analyses are largely congruent with regard to the position of Xandarellida within Artiopoda, and only differ slightly in the relationships between *Sinoburius lunaris* and other xandarellids (Fig. 9). The maximum parsimony (equal weights) analysis, not shown, was unable to resolve the relationships within Xandarellida, and resulted in a large polytomy of these taxa within Trilobitomorpha due to multiple equally parsimonious character optimizations. Maximum parsimony (implied weights) (Fig. 9a, b) recovered Xandarellida as a clade in a sister-group relationship with Nektaspida. Likewise, the implied weights analyses resolved Xandarellida + Nektaspida as sister-group to a clade comprising Conciliterga + (*Kwanyinaspis maotiashanensis* + Trilobita). The position of *Cindarella* shifted slightly between analyses with different concavity (*k*) values. *Cindarella* was recovered as either part of a clade alongside both *Luohuilinella* species (Fig. 9a), or as the earliest branching xandarellid (Fig. 9b). Implied weights consistently recovered *Sinoburius* as the sister-taxon to a clade of *Xandarella* + *Phytophilaspis* (Fig. 9a). Bayesian inference produced a less resolved overall topology for Artiopoda (Fig. 9c), but provides support for the sister-group relationship between Xandarellida + Nektaspida. Here, *Sinoburius* also forms a clade with *Phytophilaspis* and *Xandarella*, but occupies a sister-group position relative to *Xandarella* (Fig. 9c). Bayesian inference also recovered a clade comprising *Phytophilaspis* + (*Xandarella* + *Sinoburius*), which is recovered in a polytomy with *Cindarella* and both *Luohuilinella* species.

**Fig. 10:**
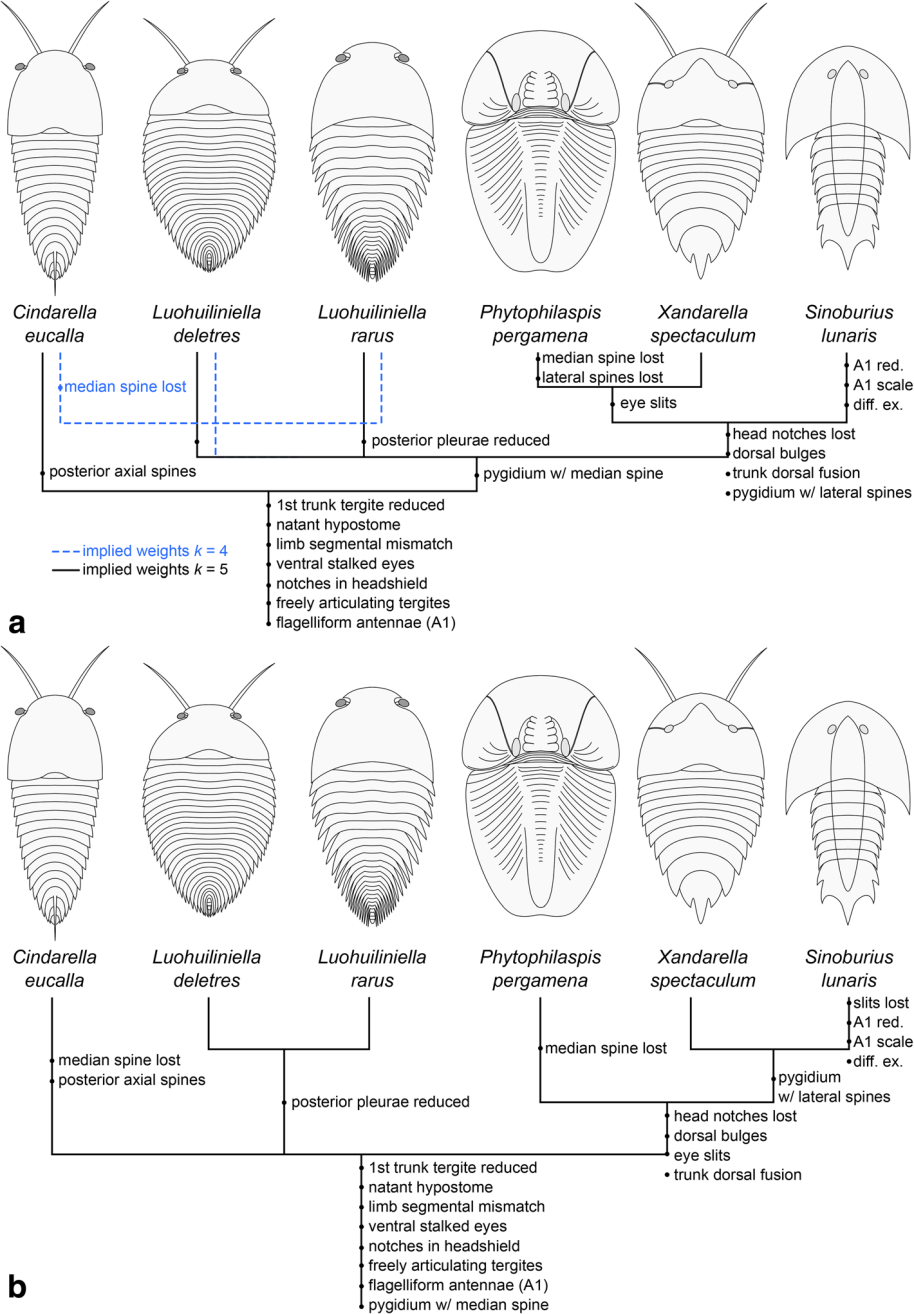
Morphological evolution of Xandarellida derived from results of phylogenetic analyses and proposed model for cephalic evolution. Note that other character optimization options are plausible, but result in either an equal or greater number of character transformations. **a** Topology based on maximum parsimony with implied weights (see Fig. 9a, b). **b** Topology based on Bayesian inference (see Fig. 9c). Abbreviations: diff. ex., differentiated exopod; red. Reduced

The results of the maximum parsimony analyses differ from previous studies in the position of *Sinoburius* within Xandarellida, and more specifically in its sister-group relationship to either *Xandarella* (Fig. 9c) or a clade comprising *Xandarella* + *Phytophilaspis* (Fig. 9b). By contrast, previous studies have recovered *Sinoburius* as either the earliest branching xandarellid [5, 6, 30, 60], as sister to a clade of *Xandarella* + *Cindarella* [32, 72], or occasionally outside of Xandarellida altogether [24, 49]. These comparisons demonstrate that the interrelationships within Xandarellida have been somewhat unstable, part of which can be attributed to the scarcity of detailed appendicular information for most representatives. Despite some variability in our results, we consistently find that *Cindarella* and *Luohuilinella* occupy either early-branching positions within Xandarellida, or a sister-group relationship to a clade consisting of the closely related *Sinoburius, Xandarella* and *Phytophilaspis*. The discrepancy between the results of the maximum parsimony and Bayesian inference analyses carry slightly different implications for the evolution and classification of Xandarellida (Fig. 10). Regardless of the topology, the presence of ventral stalked eyes, flagelliform deutocerebral antennae, natant hypostome, a reduced first trunk tergite, freely articulating tergites, and presence of limb segmental mismatch represent ancestral features within Xandarellida (Fig. 10). The presence of anterolateral head shield notches most likely represents a symplesiomorphic condition for Xandarellida, as observed in *Cindarella* and *Luohuilinella*, and secondarily lost in the clade including *Phytophilaspis, Xandarella* and *Sinoburius* (Figs. 8, 10). Depending on the position of *Cindarella* as either basal within Xandarellida or sister-group to *Luohuilinella*, the median spine in the pygidium is resolved as either autapomorphic for a clade including all taxa except for *Cindarella*, or as ancestral for Xandarellida and secondarily lost for *Cindarella* (Fig. 10a). The clade comprising *Phytophilaspis, Xandarella* and *Sinoburius* is supported by the presence of eye slits produced by the abaxial closure of the head shield around the anterolateral notches and consequent origin of the dorsal exoskeletal bulges (Fig. 8), dorsal fusion of the trunk resulting in a well-defined pygidium (Fig. 10). Whether or not the presence of lateral spines in the pygidium is ancestral for this clade depends on the position of *Phytophilaspis* relative to *Xandarella* and *Sinoburius*. In either case, the topology suggests that *Phytophilaspis* lost the pygidial spines secondarily. Likewise, the position of *Sinoburius* within this clade supports the secondary loss of eye slits, as well as the autapomorphic evolution of reduced antennae with a scale-like exite, and the substantial differentiation of the exopods on the two anterior most pairs of biramous appendages (Fig. 10). The considerable phylogenetic distance between *Sinoburius lunaris* and the nektaspidid *Misszhouia longicaudata* suggests that the presence of an antennal scale in these taxa also results from evolutionary convergence rather than this being an ancestral condition within Trilobitomorpha.

## Conclusions

The application of X-ray based computed tomography revealed novel aspects of the ventral appendicular anatomy in the Chengjiang artiopodan *Sinoburius lunaris*, and led to a comprehensive revision of the evolution and classification of this distinctive and enigmatic clade of Cambrian euarthropods. The recognition of a derived degree of appendicular tagmosis in *S. lunaris* is not only unique among trilobitomorphs, but also suggests that these early euarthropods likely possessed a more complex appendicular structure than previously considered. In this context, the use of computed tomography will prove instrumental for future studies in order to address this type of preservation, that is otherwise inaccessible through conventional imaging approaches or preparation work. Our results also clarify the evolution of archetypical characters of Xandarellida, such as the proposed origin of the eye slits as derived from the closure of the anterolateral notches in the head shield, and the phylogenetic relationships between their constituent taxa.

## Methods

This study is based on three specimens deposited at the Yunnan Key Laboratory for Palaeobiology, Yunnan University (YKLP 11407), Yuxi Normal University (YRCP 0011), and Yunnan Institute of Geological Survey (Hz-f-10-45). All specimens were collected from Ercai Village, Haikou, Kunming. Stratigraphically, they belong to the *Eoredlichia–Wutingaspis* biozone of Yu’anshan Member, Chiungchussu Formation (Cambrian Series 2, Stage 3).

The specimens were photographed under reflected light and fluorescent illumination to document details of the preserved dorsal morphology. Digital photographs were captured with a Leica DFC5000 Charge Coupled Device (CCD) attached to a Leica M205C microscope, and fluorescence microscopic images were captured with a Leica DFC7000T CCD linked to a Leica M205 FA fluorescent microscope. X-ray micro-computed tomography (CT) was used to reveal ventral structures concealed within the rock matrix. Specimen scanning was performed on a GE Phoenix Nanotom m for YRCP 0011 (voltage 110 kV, current: 100 μA) and by Zeiss X-radia 520 Versa for YKLP 11407 and Hz-f-10-45 (voltage 81 kV, current: 50 μA). Each scan generated a set of radiographs saved as TIFF stacks which were further processed with the Drishti software (version 2.4) [73]. The 3D models rendered in Drishti were screen-captured as images in the figures.

The character matrix used for the phylogenetic analyses includes 64 taxa and 89 characters. We employed an updated version of the dataset used by Du et al. [30]. The new Character 88 (antennal scale: 0, absent; 1, present) and Character 89 (head shield with lateral notches: 0, absent; 1, present) were included to better reflect the morphological diversity of *Sinoburius* and other xandarellids (see discussion). Details of all characters including new character descriptions, scorings, may be downloaded from MorphoBank [74]: 10.7934/P3437.

The Bayesian analysis was run in MrBayes 3.2 using the Monte Carlo Markov-chain model for discrete morphological characters [75, 76] for 1 million generations (four chains), with every 1000th sample stored (resulting in 1000 samples), and 25% burn-in (resulting in 750 retained samples). Convergence was diagnosed with the software Tracer [77], with effective sample size values over 200. The parsimony analyses were run in TNT [78] under New Technology Search, using Driven Search with Sectorial Search, Ratchet, Drift, and Tree fusing options activated with standard settings [79, 80]. The analysis was set to find the minimum tree length 100 times and to collapse trees after each search. All characters were treated as unordered. For comparative purposes, analyses were performed under equal and implied weights (*k* = 4 and *k* = 5) to test the effect of homoplasy penalization on the topology. Nodal support values for parsimony analyses are expressed as Group present/Contradicted frequency differences, calculated through Symmetric Resampling with 1000 replicates, each involving Traditional Search with a change of probability of 33%.

## Data Availability

The material is deposited in the Yunnan Key Laboratory for Palaeobiology, Yunnan University (YKLP 11407), Yuxi Normal University (YRCP 0011), and Yunnan Institute of Geological Survey (Hz-f-10-45). Figures supporting this article have been uploaded as part of the supplementary material. The phylogenetic data supporting the results of this article are available in the MorphoBank repository, access link 10.7934/P3437.

## Abbreviations

ans: Antennal scale
ant: Antennae
ba: Basipodite
ed: Endite
en: Endopod
ex: Exopod
ey: Eye
gn: Gnathobases
gs: Genal spine
hs: Head shield
hyp: Hypostome
lam: Lamellae
ls: Lateral spine
ms: Median spine
pd*n*: Podomere
pg: Pygidium
stn: Sternite
tc: Terminal claw
T*n*: Thoracic segment
